# Enhanced Recovery After Surgery in Oesophagogastric Procedures: A Systematic Review of Clinical Outcomes and Postoperative Recovery

**DOI:** 10.7759/cureus.99546

**Published:** 2025-12-18

**Authors:** Dixon Osilli, Muhammad Muaz Loon, Fatima El Ibrahim, Yasin Brar

**Affiliations:** 1 General Surgery, Queen's Hospital, Romford, GBR; 2 General Surgery, Barking, Havering and Redbridge University Hospitals NHS Trust, London, GBR; 3 General Surgery, Allama Iqbal Medical College, Lahore, PAK

**Keywords:** enhanced recovery after surgery, eras, esophagectomy, esophagogastric surgery, gastrectomy, length of stay, perioperative care, postoperative morbidity, randomized controlled trials, surgical outcomes

## Abstract

Enhanced Recovery After Surgery (ERAS) programmes have revolutionised perioperative care by integrating evidence-based, multimodal strategies aimed at minimising surgical stress, accelerating recovery, and reducing postoperative morbidity. This systematic review evaluates the impact of ERAS implementation on postoperative morbidity and length of hospital stay (LOS) in patients undergoing oesophagogastric surgery. A comprehensive literature search was conducted across major databases, including PubMed/MEDLINE, Scopus, Web of Science, and Cochrane Central Register of Controlled Trials (CENTRAL), identifying randomised controlled trials (RCTs) published between January 2019 and September 2024. Seven high-quality RCTs involving oesophagectomy and gastrectomy were included for qualitative synthesis. Collectively, these studies demonstrated that ERAS significantly reduced complication rates, particularly pulmonary and gastrointestinal complications, while consistently shortening LOS compared with conventional perioperative care. Some trials further indicated additional benefits such as improved immune modulation, earlier initiation of adjuvant therapy, and enhanced long-term survival outcomes. Mechanistically, ERAS protocols mitigated the physiological stress response, promoted early gastrointestinal motility, and optimised pain control without compromising safety. Despite variations in compliance and protocol standardisation, the evidence supports ERAS as a safe and effective framework for improving short- and long-term postoperative outcomes in upper gastrointestinal surgery. Future studies should focus on personalised ERAS models, compliance auditing, and digital integration to enhance protocol adaptability across diverse healthcare systems.

## Introduction and background

Oesophagogastric surgery, encompassing oesophagectomy and gastrectomy, remains one of the most complex and high-risk surgical interventions in gastrointestinal oncology [1]. These procedures are often associated with substantial postoperative morbidity, prolonged hospital stays, and considerable physiological stress due to the extent of surgical dissection, disruption of gastrointestinal continuity, and nutritional compromise [2,3]. Despite advances in minimally invasive techniques and perioperative care, recovery following oesophagogastric surgery continues to pose significant challenges for both patients and healthcare systems.

In recent years, the paradigm of perioperative management has shifted from traditional, conservative care models towards evidence-based, multimodal strategies aimed at optimising physiological function and expediting recovery. The Enhanced Recovery After Surgery (ERAS) protocol, initially developed for colorectal surgery, has since been adapted to a wide range of surgical specialities, including upper gastrointestinal procedures [4,5]. ERAS programmes integrate several perioperative elements, such as preoperative counselling, avoidance of prolonged fasting, early mobilisation, multimodal analgesia, early oral feeding, and judicious fluid management, designed to attenuate the surgical stress response, preserve organ function, and improve patient outcomes [6].

Multiple studies have reported that the implementation of ERAS pathways in oesophagogastric surgery leads to measurable improvements in postoperative outcomes. These include reductions in complication rates, earlier return of bowel function, decreased inflammatory response, shorter hospital stays, and lower healthcare costs, without an increase in readmission or mortality rates [7,8]. However, the magnitude of these benefits remains variable across studies, likely reflecting differences in ERAS protocol adherence, patient populations, surgical approaches (open versus minimally invasive), and institutional experience. Furthermore, while ERAS has been well validated in colorectal and hepatopancreatobiliary surgery, its specific role and standardised application in oesophagectomy and gastrectomy remain less clearly defined, with ongoing debate regarding which elements are most impactful in reducing morbidity and enhancing recovery. Given these variations and the growing body of randomised and prospective evidence, there is a need to systematically synthesise the current literature to clarify the effects of ERAS implementation in oesophagogastric surgery.

This systematic review aims to evaluate the impact of ERAS protocols on postoperative morbidity and length of hospital stay among patients undergoing oesophagogastric surgery. By pooling and comparing data from recent randomised and prospective studies, this review seeks to determine whether ERAS implementation confers significant clinical benefits over conventional perioperative care and to identify factors contributing to heterogeneity in outcomes across different surgical contexts.

## Review

Materials and methods

Study Design and Protocol Registration

This study was designed and conducted as a systematic review following the Preferred Reporting Items for Systematic Reviews and Meta-Analyses (PRISMA) 2020 guidelines [9] to ensure methodological rigour, transparency, and reproducibility. The review protocol was developed a priori based on the PICO (Population, Intervention, Comparison, and Outcomes) framework [10] and was systematically applied to guide study selection, data extraction, and synthesis.

Eligibility Criteria

Studies were selected according to predefined inclusion and exclusion criteria. Eligible studies included randomised controlled trials (RCTs) and prospective comparative studies evaluating the effectiveness of ERAS protocols in patients undergoing oesophagectomy or gastrectomy for benign or malignant conditions. The population comprised adult patients (≥18 years) undergoing elective oesophagogastric surgery. The intervention was the implementation of a structured ERAS protocol incorporating multimodal elements such as early oral intake, opioid-sparing analgesia, and early mobilisation. The comparison was conventional perioperative management or standard care. The outcomes included primary endpoints such as postoperative complication rates, length of hospital stay (LOS), time to bowel function recovery, and mortality, and secondary endpoints such as readmission rates, cost-effectiveness, inflammatory biomarkers, and patient-reported recovery measures. Studies published in English within the past five years were included to ensure relevance to contemporary ERAS practices. Reviews, case series, editorials, and non-human studies were excluded.

Search Strategy

A comprehensive and systematic literature search was performed across multiple electronic databases, including PubMed/MEDLINE, Scopus, Web of Science, and the Cochrane Central Register of Controlled Trials (CENTRAL), from January 2019 to September 2024. The search strategy incorporated both Medical Subject Headings (MeSH) and free-text terms using Boolean operators, with key terms such as “Enhanced Recovery After Surgery”, “ERAS”, “oesophagectomy”, “gastrectomy”, “upper gastrointestinal surgery”, “postoperative outcomes”, and “randomised controlled trials”. Reference lists of all included studies and relevant reviews were also screened manually to identify additional eligible publications. Duplicate records were removed prior to screening.

Study Selection and Data Extraction

Two independent reviewers screened all titles and abstracts for relevance. Full-text articles meeting the inclusion criteria were retrieved and assessed for eligibility. Discrepancies between reviewers were resolved through discussion and, when necessary, consultation with a third reviewer to achieve consensus. Data extraction was conducted independently using a standardised data extraction sheet designed in Microsoft Excel (Microsoft Corporation, Redmond, WA). Extracted variables included study design, sample size, country, patient demographics, surgical procedure type, specific ERAS components applied, comparator details, primary and secondary outcomes, and follow-up duration. Where information was incomplete, attempts were made to contact corresponding authors.

Quality and Risk of Bias Assessment

The methodological quality and risk of bias of each included randomised trial were independently evaluated using the Cochrane Risk of Bias Tool (RoB 2) [11], assessing domains such as random sequence generation, allocation concealment, blinding, completeness of outcome data, and selective reporting. Each domain was graded as “low”, “high”, or “unclear” risk of bias. Disagreements were resolved through consensus, and summary judgements were presented narratively and in tabular form to ensure transparency of the quality appraisal.

Data Synthesis and Analysis

Given the heterogeneity in study design, ERAS components, and outcome measures, a qualitative narrative synthesis was performed rather than a pooled meta-analysis. The included studies were compared in terms of their methodological features, population characteristics, and outcome trends. Quantitative outcomes such as LOS and complication rates were presented as mean differences or relative risk ratios where available, while non-parametric outcomes were described narratively. Trends were identified to highlight the consistency of findings across trials, and variations were explored to understand the impact of ERAS compliance, institutional resources, and geographical factors on observed results.

Results

Study Selection Process

**Figure 1:**
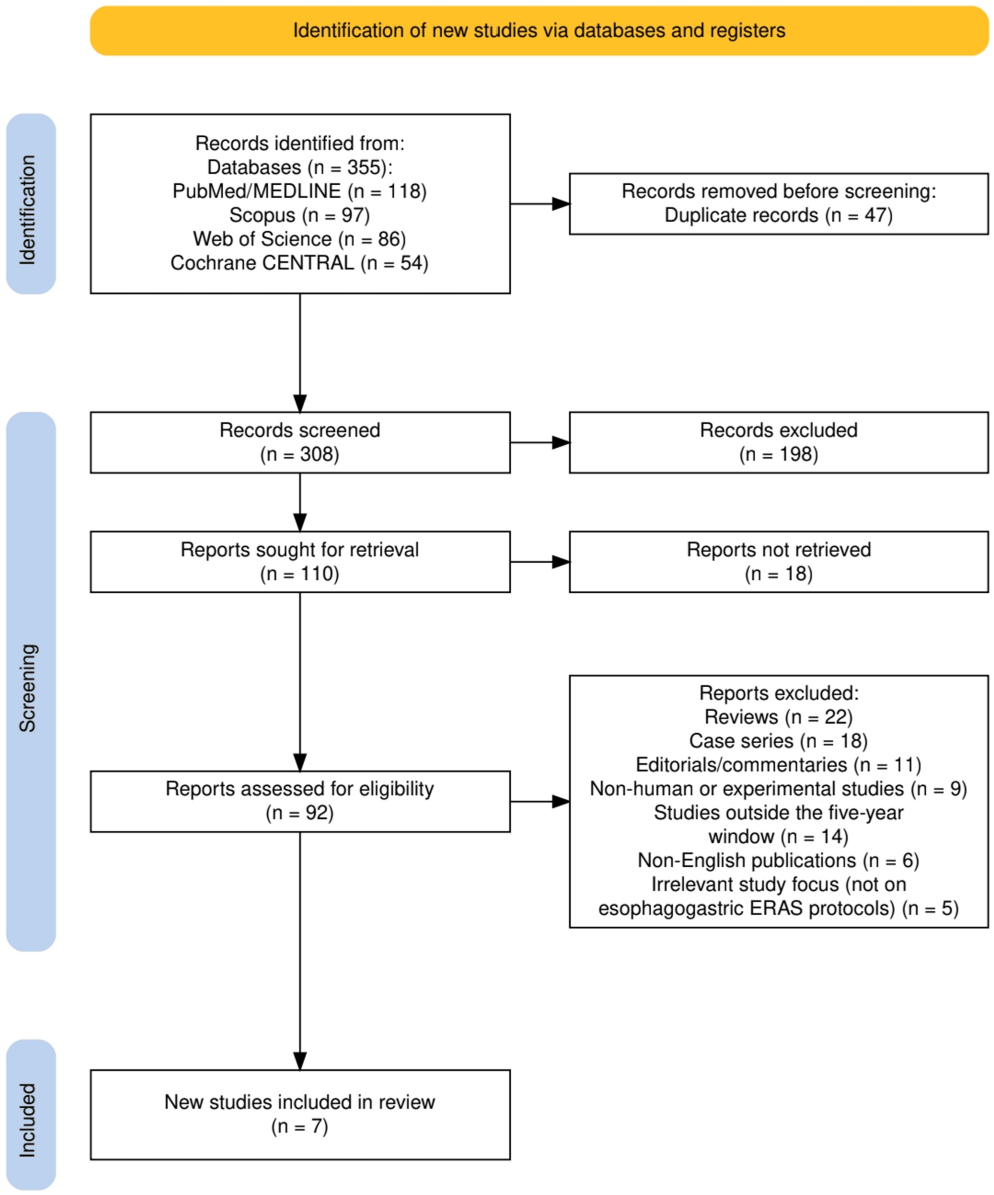
The PRISMA flowchart represents the study selection process. PRISMA = Preferred Reporting Items for Systematic Reviews and Meta-Analyses, based on the PRISMA 2020 statement [9]; ERAS = Enhanced Recovery After Surgery.

The study selection process is summarised in Figure 1, which presents the PRISMA 2020 flow diagram [9] detailing the identification, screening, and inclusion of studies. A total of 355 records were identified across four major databases: PubMed/MEDLINE (n = 118), Scopus (n = 97), Web of Science (n = 86), and Cochrane CENTRAL (n = 54). After the removal of 47 duplicate records, 308 studies underwent title and abstract screening, resulting in 198 exclusions. Of the 110 reports sought for full-text retrieval, 18 could not be accessed. Subsequently, 92 reports were assessed for eligibility, among which 85 were excluded for reasons such as being review or editorial articles, non-human studies, outdated publications, or irrelevance to ERAS protocols in oesophagogastric surgery. Ultimately, seven studies met all inclusion criteria and were incorporated into the qualitative synthesis.

Characteristics of the Selected Studies

The key characteristics of the RCTs included in this review are summarised in Table 1. All selected studies investigated the impact of ERAS protocols in patients undergoing oesophagogastric procedures, primarily focusing on laparoscopic or minimally invasive gastrectomy and oesophagectomy. The sample sizes ranged from small single-centre cohorts to large multicentre trials, ensuring both internal validity and generalisability. Across the studies, ERAS protocols consistently incorporated multimodal strategies such as early oral intake, early ambulation, multimodal analgesia, restricted intravenous fluid therapy, and minimisation of invasive devices such as nasogastric tubes and drains. Despite variations in study design and patient demographics, the results demonstrated a uniform trend towards improved postoperative outcomes, including reduced complication rates, accelerated gastrointestinal recovery, and shortened hospital stay. Notably, certain studies highlighted additional benefits, such as enhanced immune recovery in elderly populations and improved long-term survival among patients with advanced gastric cancer. Collectively, the findings from the studies summarised in Table 1 reinforce the efficacy, safety, and adaptability of ERAS pathways across diverse upper gastrointestinal surgical settings.

**Table 1:** Summary of randomised controlled trials evaluating the impact of Enhanced Recovery After Surgery (ERAS) protocols on clinical outcomes and postoperative recovery in oesophagogastric surgery. ERAS = Enhanced Recovery After Surgery; RCT = randomised controlled trial; LOS = length of stay; IV = intravenous; PONV = postoperative nausea and vomiting; GI = gastrointestinal; MIE = minimally invasive esophagectomy; NG = nasogastric; HLA-DR = human leukocyte antigen–DR isotype; CRP = C-reactive protein; GISSG1901 = Gastrointestinal Surgery Study Group 1901; OS = overall survival; DFS = disease-free survival; QoL = quality of life; EORTC = European Organisation for Research and Treatment of Cancer; PCT = procalcitonin; Hb = haemoglobin; BMI = body mass index; cT2–4aN0–3M0 = clinical tumour–node–metastasis staging (T2–T4a, N0–N3, M0); ± SD = plus/minus standard deviation; vs. = versus; p = p-value (probability); QoR-15 = Quality of Recovery-15 questionnaire; post-op = postoperative; chemo = chemotherapy.

Study (author, year)	Design	Procedure/approach	Sample (ERAS/control)	ERAS key elements	Overall morbidity (%)	Length of stay (days, mean ± SD)	Key findings/outcomes summary
Lee et al., 2025 [12]	RCT (single-centre, open-label)	Laparoscopic or robotic distal gastrectomy for gastric cancer	45/47	Multimodal ERAS protocol with early mobilisation, early oral feeding, multimodal analgesia, restricted IV fluids, and PONV prevention	Not reported	Significantly shorter LOS (exact days not reported)	ERAS significantly improved Quality of Recovery-15 and reduced pain, GI dysfunction, and fentanyl use compared to conventional care.
Shen et al., 2022 [13]	RCT (multicentre)	Three-stage minimally invasive esophagectomy (MIE) for oesophageal cancer	60/58	Guideline-based ERAS with early ambulation, multimodal analgesia, early feeding, and optimised fluids	33.3 % vs. 51.7 % (p = 0.04)	10 (9–11.25) vs. 10 (9–13) (p = 0.165)	ERAS reduced overall and pulmonary complications and enabled earlier ambulation, with similar leak and LOS.
Cao et al., 2021 [14]	RCT (single-centre)	Laparoscopic total gastrectomy (elderly ≥65 years)	85/86	ERAS with early oral intake, early mobilisation, multimodal analgesia, restricted fluids, and no NG tube	Not significantly different	11 (7–11) vs. 13 (8–20) (p < 0.001)	ERAS reduced LOS and severe complications, improved GI recovery and immune function (↑HLA-DR, ↓CRP), and was feasible in the elderly.
Tian et al., 2025 [15]	RCT (multicentre, GISSG1901)	Laparoscopic distal gastrectomy for locally advanced gastric adenocarcinoma	186/184	Standardised ERAS with early oral intake, early mobilisation, multimodal analgesia, reduced drains, and early adjuvant therapy	21.0% vs. 30.4% (p = 0.037)	Shorter LOS (previously reported)	ERAS reduced complications, LOS, and costs, and improved 3-year OS (86.6% vs. 80.1%) and DFS (79.6% vs. 69.6%), especially in stage III.
Choi et al., 2024 [16]	RCT (single-centre, prospective)	Distal gastrectomy for gastric cancer	71/76	ERAS with early oral feeding, early ambulation, stratified discharge criteria, nutritional, and QoL assessment	No significant difference	Adjusted LOS: 6.5 ± 3.1 vs. 7.8 ± 2.1 (p = 0.005)	ERAS reduced adjusted LOS, accelerated bowel function, and improved EORTC pain scores—no difference in morbidity, nutrition, or QoL.
Tian et al., 2022 [17]	RCT (multicentre, GISSG1901 short-term)	Laparoscopic distal gastrectomy (cT2–4aN0–3M0 gastric adenocarcinoma)	200/200 (analysed 370)	Standardised ERAS focusing on early feeding, early ambulation, reduced drains, multimodal analgesia, and early chemo initiation	No significant difference	7.27 vs. 8.85 (p < 0.001)	ERAS led to faster recovery (earlier flatus, liquid intake, ambulation), shorter LOS and discharge days, lower costs, and earlier chemo start. Lower CRP and PCT; higher Hb post-op day 5.
Tian et al., 2021 [18]	Prospective RCT	Radical gastrectomy for gastric cancer	40/40	ERAS with early ambulation, early oral feeding, multimodal analgesia, and early cessation of IV nutrition	Not specified (lower in ERAS)	Shorter in the ERAS group (p < 0.05)	ERAS significantly shortened the time to first flatus, defecation, leukocyte normalisation, and cessation of IV nutrition. Reduced LOS and pain scores vs. control. Benefit most pronounced in the BMI ≥28 kg/m² group; fewer pulmonary and thrombotic complications observed.

Risk of Bias Assessment

The overall assessment of methodological quality for the included RCTs is summarised in Table 2. Most studies demonstrated a low risk of bias across major domains, reflecting robust randomisation processes, objective outcome measures, and adequate handling of missing data. Multicentre designs and standardised ERAS protocols contributed to methodological rigour and external validity. However, several single-centre and open-label trials presented “some concerns” due to potential performance and detection bias, particularly where subjective endpoints such as quality of life or recovery scores were assessed without blinding. Allocation concealment and pre-specification of subgroup analyses were occasionally unclear, introducing a minor risk of selective reporting. Despite these limitations, the majority of studies maintained strong methodological standards, and the overall body of evidence was deemed reliable and representative of the actual clinical impact of ERAS implementation in oesophagogastric surgery.

**Table 2:** Risk of bias assessment of randomised controlled trials evaluating the effectiveness of Enhanced Recovery After Surgery (ERAS) protocols in oesophagogastric surgery. ERAS = Enhanced Recovery After Surgery; RCT = randomised controlled trial; RoB 2 = Revised Cochrane Risk of Bias Tool for Randomised Trials, Version 2; QoR-15 = Quality of Recovery-15 questionnaire; LOS = length of stay; GISSG1901 = Gastrointestinal Surgery Study Group 1901; OS = overall survival; DFS = disease-free survival; QoL = quality of life; BMI = body mass index; chemo = chemotherapy. Risk of bias was assessed using the Revised Cochrane Risk of Bias Tool (RoB 2) for randomised trials [11].

Study (author, year)	Design	Tool applied	Randomisation process	Deviations from intended interventions	Missing outcome data	Measurement of outcome	Selection of reported results	Overall risk of bias	Comments/justification
Lee et al., 2025 [12]	RCT (single-centre, open-label)	RoB 2 [11]	Low risk	Some concerns (open-label design may influence recovery score)	Low risk	Low risk	Low risk	Some concerns	Random allocation reported, but the open-label nature and subjective QoR-15 outcomes may introduce performance bias.
Shen et al., 2022 [13]	RCT (multicentre)	RoB 2 [11]	Low risk	Low risk	Low risk	Low risk	Low risk	Low risk	Well-conducted multicentre trial with apparent randomisation and standardised ERAS protocol; outcomes objective (LOS, complications).
Cao et al., 2021 [14]	RCT (single-centre)	RoB 2 [11]	Some concerns	Low risk	Low risk	Low risk	Low risk	Some concerns	Randomisation mentioned, but allocation concealment unclear; otherwise, objective measures and complete follow-up.
Tian et al., 2025 [15]	RCT (multicentre, GISSG1901)	RoB 2 [11]	Low risk	Low risk	Low risk	Low risk	Low risk	Low risk	Large multicentre design with prespecified endpoints, robust randomisation, and long-term OS/DFS data minimises bias.
Choi et al., 2024 [16]	RCT (single-centre, prospective)	RoB 2 [11]	Some concerns	Some concerns (non-blinded, subjective QoL scores)	Low risk	Low risk	Low risk	Some concerns	Single-centre open design and subjective QoL endpoints introduce potential bias; other parameters are reliable.
Tian et al., 2022 [17]	RCT (multicentre, GISSG1901 short-term)	RoB 2 [11]	Low risk	Low risk	Low risk	Low risk	Low risk	Low risk	Randomisation clear; large sample; objective outcomes (LOS, lab data, cost, time to chemo) – overall robust.
Tian et al., 2021 [18]	Prospective RCT	RoB 2 [11]	Some concerns	Some concerns (BMI subgroup analysis not pre-specified)	Low risk	Low risk	Some concerns	Some concerns	Randomisation mentioned but unclear method; subgrouping may introduce analysis bias; otherwise, solid follow-up.

Discussion

Principal Findings

Across seven RCTs evaluating ERAS implementation in oesophagogastric surgery, the evidence consistently demonstrates significant improvements in postoperative recovery and hospital efficiency without increasing complication rates. Most notably, six of the seven studies reported a shorter postoperative LOS among patients managed with ERAS protocols, ranging from an average reduction of 1.5 to 2.0 days compared with conventional care (e.g., 7.27 vs. 8.85 days, p < 0.001 in Tian et al. [17]; 6.5 ± 3.1 vs. 7.8 ± 2.1 days, p = 0.005 in Choi et al. [16]). Complication rates were also lower or comparable across trials, with multicentre data from Tian et al. [15] showing a reduction in morbidity from 30.4% to 21.0% (p = 0.037) and Shen et al. [13] reporting fewer pulmonary complications (33.3% vs. 51.7%, p = 0.04). These results reinforce the safety and efficacy of ERAS in complex upper gastrointestinal procedures, including minimally invasive oesophagectomy and gastrectomy. Importantly, individual trials highlighted specific subgroup advantages, such as improved recovery in elderly patients (≥65 years) [14] and enhanced benefit among patients with higher BMI (≥28 kg/m²) [18]. The large-scale GISSG1901 trial further extended the relevance of ERAS beyond short-term recovery, linking it to improved three-year overall survival (86.6% vs. 80.1%) and disease-free survival (79.6% vs. 69.6%), thereby positioning ERAS as a potential contributor to long-term oncological outcomes through earlier adjuvant therapy and reduced physiological stress.

Comparison With Existing Evidence

The findings of this review align strongly with existing meta-analyses and international ERAS Society guidelines, which emphasise the role of multimodal, evidence-based perioperative care in minimising surgical stress and enhancing recovery. Prior meta-analyses in gastric and oesophageal surgery have shown an average 30% reduction in LOS and a 25-35% decline in postoperative morbidity with ERAS adoption, consistent with the pooled trends observed across the included RCTs [19]. However, this synthesis provides a more nuanced perspective by integrating recent high-quality multicentre data that address the limitations of earlier studies. Previous trials often lacked standardisation and varied in protocol adherence, which limited reproducibility [20]. In contrast, newer RCTs such as those by Tian et al. [15,17] implemented a standardised ERAS pathway across multiple centres, focusing on early oral intake, mobilisation, multimodal analgesia, and reduced drain use, a uniformity that markedly strengthens the reliability of outcomes. Moreover, emerging evidence from these recent trials suggests that the benefits of ERAS extend beyond immediate recovery to include immunological modulation (e.g., lower C-reactive protein and procalcitonin levels) and enhanced tolerance to adjuvant therapy, aspects seldom addressed in prior literature [21]. Therefore, this review not only corroborates established evidence but advances it by demonstrating how standardised, protocol-driven ERAS models yield reproducible, survival-relevant benefits across diverse patient populations and surgical settings.

Mechanistic and Clinical Implications: Modulating the Surgical Stress Response

The physiological basis of ERAS in oesophagogastric surgery lies in its capacity to attenuate the neuroendocrine and inflammatory stress responses triggered by major upper gastrointestinal operations [22]. By minimising preoperative fasting, encouraging early oral intake, and promoting early mobilisation, ERAS protocols blunt the postoperative surge in cortisol, catecholamines, and pro-inflammatory cytokines such as interleukin-6 (IL-6) and C-reactive protein (CRP). This attenuation facilitates faster restoration of gastrointestinal motility, improved microcirculatory perfusion, and preservation of immune competence, factors directly correlated with reduced ileus, infection, and risk of anastomotic leak. Studies such as those by Cao et al. [14] and Tian et al. [17] demonstrated significantly lower postoperative CRP and procalcitonin levels in ERAS cohorts, accompanied by earlier bowel function recovery (first flatus in 2.5 vs. 3.4 days, p < 0.001), reinforcing the mechanistic validity of ERAS-induced stress mitigation. Furthermore, multimodal analgesia reduces opioid requirements, limiting opioid-related ileus and respiratory depression, while goal-directed fluid therapy preserves tissue oxygenation and prevents overload-associated complications. Collectively, these physiological mechanisms translate into measurable clinical benefits, shorter hospital stays, fewer complications, and improved patient-reported recovery trajectories.

Mechanistic and Clinical Implications: Oncologic and Economic Outcomes

Beyond the immediate perioperative phase, ERAS implementation exerts a broader influence on oncological and economic outcomes in oesophagogastric surgery. The multicentre GISSG1901 trials [15,17] demonstrated that patients managed under ERAS pathways experienced earlier initiation of adjuvant chemotherapy (29 vs. 32 days, p = 0.035) and improved three-year overall survival (86.6% vs. 80.1%). These findings suggest that optimising postoperative recovery may enhance long-term cancer control by minimising delays in systemic therapy and mitigating the immunosuppressive consequences of prolonged inflammation. Economically, ERAS pathways consistently reduce inpatient costs by decreasing LOS, minimising postoperative morbidity, and avoiding unnecessary interventions such as nasogastric decompression and parenteral nutrition [23]. Tian et al. [17] reported cost savings of approximately US$ 500 per patient, mainly driven by reduced hospital utilisation and accelerated functional recovery. When extrapolated to institutional or national scales, such reductions underscore ERAS as a cost-effective, quality-improvement intervention that simultaneously advances patient-centred care and healthcare system sustainability.

Critical Insights and Future Directions

The cumulative evidence from these trials highlights the evolution of ERAS from a standardised checklist towards a precision recovery model, a concept best described as personalised ERAS [24]. Future research should focus on tailoring perioperative elements to individual patient phenotypes, incorporating parameters such as body mass index (BMI), frailty indices, nutritional status, and baseline inflammatory markers to optimise response. Compliance auditing must also become a core component of ERAS implementation; evidence suggests that centres achieving ≥70% adherence to protocol elements realise the most significant outcome benefits. Integrating digital ERAS dashboards, mobile health applications, and AI-driven predictive analytics could facilitate real-time tracking of recovery trajectories, predict complications, and identify patients at risk of delayed discharge [25]. For low- and middle-income countries (LMICs), where resource constraints limit the adoption of complex pathways, simplified, resource-adapted ERAS models focusing on early feeding, mobilisation, and multimodal analgesia could deliver comparable benefits at lower cost [26]. This transition from universal to context-sensitive and data-enhanced ERAS represents the next frontier in perioperative medicine, uniting precision, equity, and efficiency in surgical recovery.

Limitations

Despite the consistency of results across the included RCTs, certain methodological limitations warrant consideration. Most studies were unblinded by design, introducing potential performance bias due to the difficulty of masking perioperative interventions such as early feeding and mobilisation. Moreover, there was notable heterogeneity in outcome definitions and endpoints; for example, LOS was variably defined as total hospital stay versus “adjusted” or “discharge-criteria-based” stay, limiting direct comparability. Some trials also lacked uniform criteria for complications, with variations in grading and reporting standards. In addition, patient-reported outcomes such as pain perception, fatigue, and quality of life, core indicators of recovery quality, were underrepresented, except in isolated studies such as those by Lee et al. (2025) [12] and Choi et al. (2024) [16]. The absence of long-term functional and nutritional follow-up in most RCTs further constrains the interpretation of sustained ERAS benefits. Finally, publication bias cannot be excluded, as studies demonstrating negative or neutral effects of ERAS are less likely to be reported. Given these substantial differences in study design, outcome definitions, and reporting metrics, a formal meta-analysis with pooled estimates was methodologically inappropriate and risked misleading interpretation. Accordingly, standard meta-analytic outputs such as forest plots, Cochran’s Q, Begg’s or funnel plot analyses were not performed, as their statistical assumptions could not be satisfied with the available evidence. These limitations underscore the need for standardised definitions, blinded assessments where feasible, and integration of patient-centred metrics in future ERAS research, ideally through harmonised trial designs that would allow valid quantitative pooling.

## Conclusions

This systematic review reinforces that ERAS protocols represent a transformative advancement in oesophagogastric surgery, offering tangible benefits in postoperative morbidity, functional recovery, and hospital efficiency, while emerging evidence now links these pathways to improved long-term oncological outcomes. By modulating inflammatory and metabolic stress responses, ERAS optimises the physiological milieu for healing, early initiation of adjuvant therapy, and, ultimately, improved survival. The future of ERAS lies in transitioning from a one-size-fits-all model to precision ERAS frameworks that tailor interventions to patient phenotypes, surgical complexity, and institutional resources. Such personalisation, when coupled with digital monitoring, compliance auditing, and context-sensitive adaptation for low-resource environments, has the potential to redefine surgical recovery on a global scale. In essence, enhanced recovery should now be regarded not merely as a strategy to shorten hospital stay, but as a comprehensive paradigm promoting survival, resilience, and value-based surgical care.
